# Lethal Mutagenesis of RNA Viruses and Approved Drugs with Antiviral Mutagenic Activity

**DOI:** 10.3390/v14040841

**Published:** 2022-04-18

**Authors:** Ikbel Hadj Hassine, Manel Ben M’hadheb, Luis Menéndez-Arias

**Affiliations:** 1 Unité de Recherche UR17ES30 “Génomique, Biotechnologie et Stratégies Antivirales”, Institut Supérieur de Biotechnologie, Université de Monastir, Monastir 5000, Tunisia; hadj_hassine_ekbell@yahoo.fr (I.H.H.); benmhadhebmanel@yahoo.fr (M.B.M.); 2 Centro de Biología Molecular “Severo Ochoa” (Consejo Superior de Investigaciones Científicas & Universidad Autónoma de Madrid), 28049 Madrid, Spain

**Keywords:** lethal mutagenesis, error catastrophe, nucleoside analogs, ribavirin, favipiravir, molnupiravir, RNA polymerase, SARS-CoV-2, HIV

## Abstract

In RNA viruses, a small increase in their mutation rates can be sufficient to exceed their threshold of viability. Lethal mutagenesis is a therapeutic strategy based on the use of mutagens, driving viral populations to extinction. Extinction catastrophe can be experimentally induced by promutagenic nucleosides in cell culture models. The loss of HIV infectivity has been observed after passage in 5-hydroxydeoxycytidine or 5,6-dihydro-5-aza-2′-deoxycytidine while producing a two-fold increase in the viral mutation frequency. Among approved nucleoside analogs, experiments with polioviruses and other RNA viruses suggested that ribavirin can be mutagenic, although its mechanism of action is not clear. Favipiravir and molnupiravir exert an antiviral effect through lethal mutagenesis. Both drugs are broad-spectrum antiviral agents active against RNA viruses. Favipiravir incorporates into viral RNA, affecting the G→A and C→U transition rates. Molnupiravir (a prodrug of β-d-N^4^-hydroxycytidine) has been recently approved for the treatment of SARS-CoV-2 infection. Its triphosphate derivative can be incorporated into viral RNA and extended by the coronavirus RNA polymerase. Incorrect base pairing and inefficient extension by the polymerase promote mutagenesis by increasing the G→A and C→U transition frequencies. Despite having remarkable antiviral action and resilience to drug resistance, carcinogenic risks and genotoxicity are important concerns limiting their extended use in antiviral therapy.

## 1. Quasispecies and Lethal Mutagenesis

Many viruses evolve rapidly [1]. Their compact genomes, high mutation rates, short replication times, and large population sizes are responsible for the generation of highly variable populations forming mutant swarms known as viral quasispecies [1]. A viral quasispecies refers to a population structure containing a large number of variant genomes with nonidentical, but closely related, mutant and recombinant genomes [2]. Also known as mutant spectra, mutant swarms, or mutant clouds, their composition and dynamics are continuously changing as viral replication and selection proceed. Mutant spectra containing related minority variants at low frequencies constitute phenotypic reservoirs and are well suited to respond to changing environments, a trait that is most relevant to viral populations. 

The quasispecies concept was introduced by Eigen and Schuster in the 1970s to explain the self-organization and evolution of primitive RNA (or RNA-like) replicons when these molecules emerged from a mixture of prebiotic chemicals on the early Earth and began to replicate at the onset of life [3,4]. The existence of RNA virus quasispecies was independently demonstrated after studying the evolution of *Escherichia coli* bacteriophage Qβ RNA using a cell-free system [5]. The bacteriophage Qβ RNA is a small, single-stranded (ss)RNA that replicates through a double-stranded (ds)RNA intermediate. Qβ RNA can be replicated autocatalytically in vitro, while the sequence and composition of newly synthesized products depend on the fidelity of the Qβ replicase, nucleotide pools, metal cations, temperature, and speed of replication [6,7]. The mutation rates calculated for phage Qβ populations were estimated at around 10^−4^ per copied nucleotide, well above the rates calculated for DNA viruses and microorganisms [8,9].

Error catastrophe could be defined as a cumulative loss of genetic information in a lineage of organisms due to high mutation rates. The process leading to viral extinction through the accumulation of errors is known as lethal mutagenesis. Error catastrophe occurs when the mutation rate exceeds an error threshold. Viruses and bacteria have evolved to maintain a characteristic error rate. RNA viruses have very high mutation rates and replicate near the error threshold for the maintenance of genetic information [10]. A modest 1.1- to 2.8-fold increase in their mutation frequency can be sufficient to enter error catastrophe, as shown for vesicular stomatitis virus and poliovirus [11]. 

**Figure 1:**
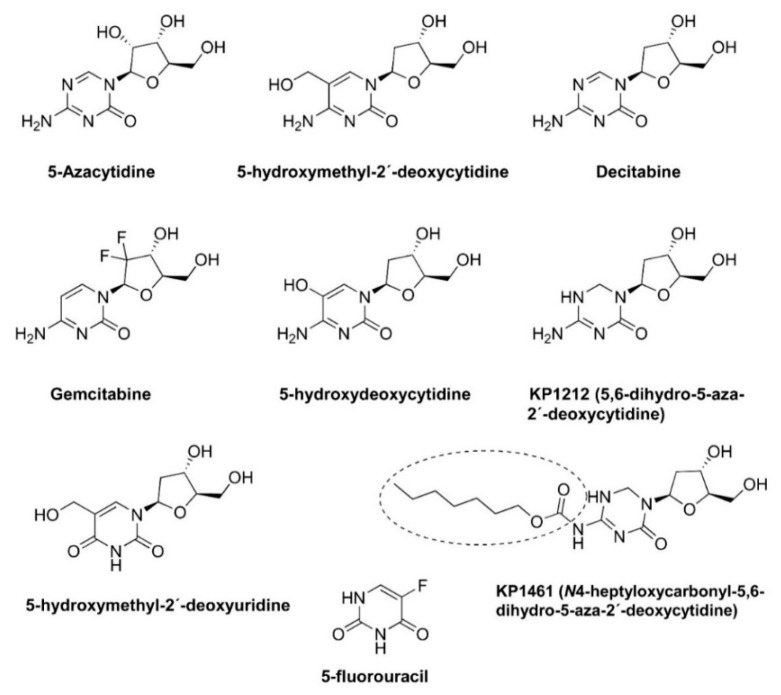
Chemical structures of mutagenic base and nucleoside analogs and prodrugs used in lethal mutagenesis experiments. KP-1461 is a prodrug of KP-1212.

Lethal mutagenesis is an antiviral strategy that aims at extinguishing a virus by increasing the viral mutation rates during replication, usually through the use of mutagenic nucleoside analogs [12] (Figure 1).

**Figure 2:**
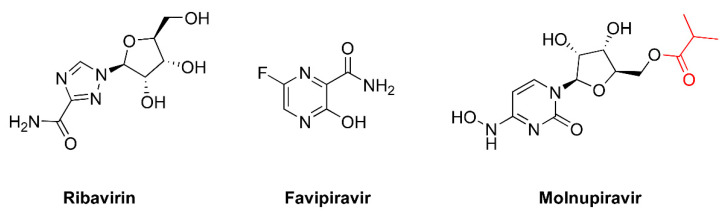
Chemical structures of approved drugs inducing mutagenesis in viral replication assays. Molnupiravir is the isopropylester prodrug of β-d-N^4^-hydroxycytidine. The isopropylester moiety is shown in red.

The term ‘lethal mutagenesis’ was coined in 1999 by Loeb and colleagues after showing that exposing the human immunodeficiency virus type 1 (HIV-1) LAI strain to 1 mM 5-hydroxydeoxycytidine (Figure 1) led to the loss of viral titer after 24 sequential passages in cell cultures [13]. The sequencing of part of the HIV-1 reverse transcriptase (RT) coding region of the penultimate passage prior to extinction revealed 2.6- and 5-fold increases in the frequency of A-to-G transitions (A→G) in two separate experiments [13]. These results stimulated further studies addressing the potential of mutagenic nucleoside analogs as antiviral agents driving viral populations to extinction. The other compounds shown in Figure 1 have been tested in HIV-1 and other RNA viruses, as well as in hepatitis B virus, with variable efficacies. In addition, virological and biochemical studies have demonstrated that the antiviral effect of approved drugs, such as ribavirin, favipiravir, and, more recently, molnupiravir, is due (at least in part) to their mutagenic action. Their chemical structures are shown in Figure 2. In this review, we discuss these studies and their implications for the further improvement of this antiviral strategy.

## 2. Mutation Rates and Fidelity of Viral Polymerases 

The mutation rates vary considerably among viruses [14,15]. While DNA viruses exhibit mutation rates ranging between 10^−8^ and 10^−6^ substitutions per nucleotide per cell infection, RNA viruses have mutation rates in the order of 10^−6^ to 10^−4^ [14,16]. A high replication rate combined with a high mutation rate allows RNA viruses to explore the sequence space and evade the immune system, even in situations where the majority of the viral progenies are nonviable [17]. 

Reverse-transcribing RNA viruses, such as retroviruses, use the viral RT to convert their ssRNA genomes into dsDNA, which is then integrated into the host genome and replicated along with it by eukaryotic DNA polymerases [18]. The mutation rates caused by the inactivation of a reporter gene range from 2 × 10^−5^ to 6 × 10^−6^ per nucleotide and replication cycle for many retroviruses, such as spleen necrosis virus, Rous sarcoma virus, murine leukemia virus (MLV), bovine leukemia virus, HIV-1, and human T-cell leukemia virus I (reviewed in [19]). O’Neil et al. [20] reported a mutation rate for HIV-1 of 8.5 × 10^−5^ mutations per base pair and replicative cycle, based on the variability observed at the long terminal repeats of the viral genome. In their study, the authors concluded that HIV-1 mutagenesis results from nucleotide misincorporation by the viral RT, although some contribution of the host RNA polymerase cannot be excluded. 

Retroviral RTs lack a proofreading 3′–5′ exonuclease domain and have a relatively high propensity to extend mispaired 3′ ends when synthesizing viral DNA (reviewed in [21]). In addition, viral and host cells may also contribute to the observed variability in different retroviruses. Thus, feline immunodeficiency virus (FIV), equine infectious anemia virus (EIAV), mouse mammary tumor virus (MMTV), and Mason-Pfizer money virus (MPMV) encode a dUTP pyrophosphatase (dUTPase) within their genomes [22,23]. These enzymes reduce the mutation levels by preventing the incorporation of uracil into the viral genome, thereby safeguarding efficient reverse transcription [24]. In HIV-1, single-cycle replication assays using the *lacZα* gene as a mutational target showed that the deletion of the *vpr* gene produced a four-fold increase in the viral mutation rate [25]. On the other hand, apolipoprotein B mRNA-editing, catalytic polypeptide-like enzymes (APOBEC3) are cytidine deaminases of the host cell that cause hypermutations of nascent retroviral genomes by the deamination of cytidine residues [26,27]. APOBEC proteins are encapsidated within the virion, but the viral protein Vif suppresses their mutagenic effects by promoting APOBEC degradation in the ubiquitin-proteasome pathway (for a review, see [28]).

The HIV-1 RT is a heterodimer composed of subunits of 66 and 51 kDa. The large subunit contains an RNase H domain at its C-terminal end that is absent from p51. The RNase H activity of the HIV-1 RT degrades the RNA strand in the RNA/DNA duplexes formed during reverse transcription (for a review, see [18]). The DNA polymerase active site of the enzyme is located in the 66-kDa subunit and contains the catalytic residues Asp110, Asp185, and Asp186. The polymerase domain shares a subdomain arrangement found in many polymerases consisting of fingers, palm, and thumb subdomains, and a series of conserved motifs (A, B, C, D, and E) contributing key residues of the nucleotide-binding site [18]. In the fingers subdomain of p66, the incoming dNTP is tightly coordinated by the side-chains of Lys65 and Arg72, the main chain amido groups of Asp113 and Ala114, and two magnesium cations. Tyr115, Phe116, and Gln151 are additional residues delineating the dNTP binding pocket. Site-directed mutagenesis studies have shown that Lys65 has a major influence on the fidelity of HIV-1 RTs. Its substitution by Arg rendered enzymes with increased fidelity of DNA synthesis in HIV-1 M and O strains [29,30]. On the other hand, the substitution of Ala for Tyr115 conferred a mutator phenotype, as demonstrated by using enzymatic and cell-based assays [31,32,33].

HIV and other retroviruses evolve at rates similar to those of non-reverse transcribing RNA viruses (often referred to as riboviruses). Riboviral RNA-dependent RNA polymerases (RdRps) share the classical fingers–palm–thumb subdomain arrangement and lack 3′ exonuclease proofreading activity [34,35]. Notable exceptions are members of the Nidovirales order, including coronaviruses, a family of positive-strand RNA viruses encoding an RdRp complex (nsp7/(nsp8)_2_/nsp12) that associates with a protein subunit (nsp14) with 3′ exonuclease activity (for recent reviews, see [36,37]). The coronavirus genomes are among the largest known for RNA viruses, ranging from ∼26–32 kbp [38]. The amino-terminal half of SARS-CoV nsp14 (59 kDa) contains active site residues (Asp90, Glu92, Glu191, Asp273, and His268) also found in the cellular enzymes of the DEDD superfamily, including those that catalyze DNA proofreading. The substitution of Ala for Asp90 or Glu92 in SARS-CoV and the equivalent positions of murine hepatitis virus (MHV) rendered viable mutants that showed 15- to 20-fold increases in mutation rates, and were up to 18 times greater than those tolerated for fidelity mutants of other RNA viruses [39,40].

## 3. Driving HIV into Error Catastrophe and Preliminary Clinical Development of KP1212/KP1461 as an Antiretroviral Agent Causing Lethal Mutagenesis

Available evidence in the late 1990s suggested that a small increase in the mutation rate of HIV could lead to error catastrophe and viral extinction [11]. Nucleoside analogs (e.g., 3′-azidothymidine (AZT), 5-azacytidine, and 5-hydroxydeoxycytidine) (Figure 1) have been shown to effectively increase the mutation rates of several retroviruses, including spleen necrosis virus, MLV, FIV, and HIV-1 [41,42,43,44,45] (Table 1). These effects were consistent with an observed increase in the frequencies of G→C transversions (5-azacytidine) or G→A transitions (3′-azidothymidine). Interestingly, 5-azacytidine promotes G→C hypermutagenesis in HIV-1, but not in oncoretroviruses (e.g., MLV or feline leukemia virus) [46]. The mutagenic effect is enhanced by the conversion of 5-azacytidine into 5-aza-2′-deoxycytidine, which promotes G→C transversion during reverse transcription [47]. However, enzymatic assays have shown that, unlike the HIV-1 RT, the MLV RT is able to incorporate 5-aza-2′-deoxycytidine-triphosphate while inhibiting the polymerization reaction by introducing pause sites and reducing the amount of fully extended product [46].

**Table 1:** Mutagenic nucleosides and their mutational effects on HIV-1.

Mutagenic Nucleoside	Increase in Mutation Frequency	Mutational Preference	References
5-azacytidine	2.3-fold	G/C transversions	[45,47]
5-fluorouracil	<1.5-fold	A/G, U/C transitions	[48]
5-hydroxymethyl-2′-deoxycytidine	3.4-fold	G→A, G→T	[49]
5-hydroxymethyl-2′-deoxyuridine	3.1-fold	A→G, G→C	[49]
Decitabine (5-aza-2′-deoxycytidine)	4.1-fold	G/C transversions	[50]
Gemcitabine (2,2(′)-difluoro-2(′)-deoxycytidine)	<1.5-fold		[48]

As of today, nucleoside analogs were widely used in the late 1990s for the treatment of HIV infection [51,52]. In this context, five nucleosides were initially tested as potential lethal mutagenesis agents against HIV-1: 5-hydroxydeoxycytidine, O^4^-methylthymidine, O^6^-methyldeoxyguanosine, 8-aminodeoxyguanosine, and 8-oxodeoxyguanosine [13]. These nucleoside analogs were selected because they were phosphorylated in human cells and then incorporated into DNA by HIV-1 RT while being resistant to the action of host cell DNA repair systems. Of course, a very important property of these molecules is their ability to generate mismatched base pairs when incorporated into DNA. Mismatches are commonly due to the tautomerization of bases during DNA replication and result in the generation of mutations. 

Viral extinction associated with increased mutagenesis was observed only in the experiments carried out with 5-hydroxydeoxycytidine [13]. However, hypermutation was detected, but only in a few clones passaged in the presence of O^4^-methylthymidine. Although this experiment provided a proof of principle for lethal mutagenesis, the results were not easily reproduced, probably due to the strict experimental requirements introduced in those experiments. Thus, Tapia et al. [53] were unable to extinguish the high-fitness HIV-1 isolate F96 after 16 serial passages in peripheral mononuclear cells (PBMCs) or MT-4 cells in the presence of 2 mM 5-hydroxydeoxycytidine. However, systematic extinction of HIV-1 was observed when a combination of the mutagenic agent (5-hydroxydeoxycytidine) and the antiretroviral drug 3′-azido-3′-deoxythymidine (AZT) was used. Despite the low concentration of AZT (0.01 µM) used in these experiments, extinction due to the expected mutagenic effect of 5-hydroxydeoxycytidine was not demonstrated, since the mutation frequencies in pre-extinction passages remained unchanged. At sublethal doses, AZT has no significant effect on frameshifts and most base substitution mutations. However, recent studies showed that AZT and other chain-terminating nucleoside RT inhibitors (e.g., 2′-3′-didehydro-3′-deoxythymidine (stavudine) and 2′-3′-dideoxyinosine (didanosine)) are mutagenic for template-switch-generated genetic mutations [54].

The screening of potential mutagenic nucleosides led to the selection of 5,6-dihydro-5-aza-2′-deoxycytidine (KP1212) (Figure 1), another deoxycytidine analog that extinguished HIV in culture after 13 passages [55]. KP1212, and its prodrug KP1461 (Figure 1), are prototypes of a new class of antiretroviral drugs designed to increase the viral mutation rates. The cloning and sequencing of HIV-1 RT-coding regions and the V3 loop in the envelope gene from the eleventh passage indicated that KP1212 at 10 µM enhanced the mutation frequencies by 1.5- and 1.9-fold, respectively [12,55]. A→G and C→T transitions were the most frequent mutations. These observations were consistent with the existence of a broad ensemble of interconverting tautomers of KP1212, among which enolic forms dominated [56]. It was found that KP1212 paired with both A (10%) and G (90%), in agreement with data obtained in cell culture experiments. KP1461 entered clinical trials, and, at phase 2, individuals previously treated with antiretroviral drugs received 1600 mg of KP1461 twice per day for 124 days. As expected, KP1461 treatment resulted in a significant increase in transition mutations, especially G→A and A→G [57]. KP1416 was safe and well-tolerated, but its development into an antiretroviral drug was halted due to inconsistent antiviral activity. Further attempts to improve its efficiency still remain at an investigational level.

HIV mutagenesis can be also modulated by ribonucleotide reductase inhibitors, such as hydroxyurea or resveratrol [58]. These compounds are small nonnucleosidic molecules that alter the dNTP pools, therefore interfering with polymerase fidelity. Their effects in mutagenesis have been investigated in experiments using gemcitabine [2,2(′)-difluoro-2(′)-deoxycytidine] and clofarabine [(2′S)-2′-deoxy-2′-fluoro-2-chloro-adenosine] as deoxynucleoside analogs (Figure 1) [58,59,60,61]. It has been noted that 5-azacytidine has anti-HIV-1 activity (i.e., EC_50_ around 200 nM) and low cytotoxicity, and it has been shown to act in synergy with gemcitabine and resveratrol [62,63]. The EC_50_ values obtained for HIV-2 inhibition with 5-azacytidine, clofarabine, and resveratrol were significantly lower than those obtained with HIV-1 [64]. These findings were consistent with the higher susceptibility of HIV-2 RT to dNTP pool alterations [18,65] and suggest that HIV-2 is more susceptible to lethal mutagenesis, particularly when the antiviral agent is combined with a ribonucleotide reductase inhibitor, such as resveratrol. In addition, studies carried out in HIV-1 cell cultures have shown that gemcitabine or resveratrol promote the mutagenic activity of KP1212 by increasing the frequency of G→C transversions [59].

As in retroviruses, *Hepadnaviridae* replicate through the reverse transcription of an RNA intermediate. Experiments in vitro have shown that 5-aza-2′-deoxycytidine and 5-azacytidine are able to eliminate hepatitis B virus transfer to target cells. Both nucleosides induced mutagenesis during relaxed circular DNA (rcDNA) formation while reducing the amount of viral DNA synthesis during rcDNA formation and the conversion of rcDNA to covalently closed circular DNA (cccDNA) [66]. Consistent with HIV-1, in these studies, an antiviral drug synergy was also observed between 5-aza-2′-deoxycytidine or 5-azacytidine and gemcitabine.

## 4. Lethal Mutagenesis in Non-Retroviral RNA Viruses: An Overview of Studies Showing the Effects of Base and Nucleoside Analogs

The introduction of lethal mutagenesis as a plausible antiviral strategy led researchers to test whether known mutagenic nucleosides and available antiviral drugs could promote mutagenesis in many different viruses. RNA viruses show high mutation rates and are particularly sensitive to the genetic meltdown expected by the increase in the mutation frequencies. As mentioned above, early work by Holland and colleagues using polioviruses and vesicular stomatitis virus showed that a modest increase (1.1- to 2.8-fold) in the mutation frequency would be sufficient to decrease viral titers more than 100-fold [11,67]. Since then, several mutagenic nucleoside analogs have been tested in cell culture and animal models in order to evaluate their antiviral efficacy and mechanism of action. 

Numerous studies have shown that single-stranded RNA viruses can be extinguished by increasing their mutation rate during replication using different base and nucleoside analogs (Table 2). However, we are not aware of similar studies carried out with double-stranded RNA viruses (e.g., reoviruses, birnaviruses, and others). In general, 5-fluorouracil (Figure 1), ribavirin, favipiravir, and, more recently, β-d-N^4^-hydroxycytidine (molnupiravir) (Figure 2) appear as the most effective compounds inducing lethal mutagenesis. The last three drugs have been approved for clinical use in different countries. Although they have broad-spectrum activity, there are important concerns about their efficacy and side effects due to their mutagenic properties. Thus, Zhou et al. [68] recently demonstrated that treatment with β-d-N^4^-hydroxycytidine can induce mutations in the host cell DNA. Inside the cell, ribonucleotide reductases catalyze the conversion of β-d-N^4^-hydroxycytidine to the 2′-deoxyribonucleotide form than can be then incorporated into DNA while inducing mutagenesis in the host. Therefore, the development of reliable genotoxicity assays will be critical for the establishment of lethal mutagenesis as an antiviral strategy.

**Table 2:** Studies showing lethal mutagenesis in single-stranded RNA viruses grown in the presence of mutagenic base and nucleoside analogs.

Virus Names		Family/Genus	Mutagenic Base and Nucleoside Analogs	Refs.
**Positive-strand RNA viruses**				
-	Poliovirus	*Picornaviridae/Enterovirus*	Ribavirin, 5-nitrocytidine, 6-(β-d-ribofuranosyl)-3,4-dihydro-8H-pyrimido [4,5-c][1,2]oxazin-7-one, and *N*-6-substituted purine analogs (JA28 and JA30)	[69,70,71,72]
	Coxsackievirus	*Picornaviridae/Enterovirus*	Ribavirin, and *N*-6-substituted purine analogs	[72,73]
	Encephalomyocarditis virus	*Picornaviridae/Cardiovirus*	5-Fluorouracil	[74]
	Foot-and-mouth disease virus	*Picornaviridae/Aphthovirus*	5-Fluorouracil, ribavirin, and favipiravir	[74,75,76,77,78,79]
	Murine norovirus	*Caliciviridae/Norovirus*	Favipiravir	[80]
	Dengue virus	*Flaviviridae/Flavivirus*	3-Hydroxy-2-pyrazinecarboxamide (T-1105), and its ribose derivative (T-1106)	[81]
	Usutu virus	*Flaviviridae/Flavivirus*	5-Fluorouracil and favipiravir, while ribavirin effects are less pronounced.	[82]
	West Nile virus	*Flaviviridae/Flavivirus*	Ribavirin and favipiravir	[83,84]
	Zika virus	*Flaviviridae/Flavivirus*	Ribavirin and favipiravir	[82]
	GB virus B	*Flaviviridae/Hepacivirus*	Ribavirin	[85]
	Hepatitis C virus	*Flaviviridae/Hepacivirus*	Ribavirin and favipiravir	[86,87,88,89,90,91,92]
	Hepatitis E virus	*Hepeviridae/Orthohepevirus*	Ribavirin	[93]
	Venezuelan equine encephalitis virus	*Togaviridae/Alphavirus*	β-d-*N*^4^-hydroxycytidine (molnupiravir)	[94]
	SARS-CoV-2	*Coronaviridae/Betacoronavirus*	Favipiravir and β-d-*N*^4^-hydroxycytidine (molnupiravir)	[68,95,96]
	Tobacco mosaic virus	*Virgaviridae/Tobamovirus*	5-Fluorouracil	[97]
**Negative-strand RNA viruses**				
	Influenza A virus	*Orthomyxoviridae/* *Alphainfluenzavirus*	Ribavirin, 5-azacytidine, 5-fluorouracil, and β-d-*N*^4^-hydroxycytidine (molnupiravir)	[98,99]
	Vesicular stomatitis virus	*Rhabdoviridae/Vesiculovirus*	5-Fluorouracil	[74,100]
	Hantaan virus	*Hantaviridae/Orthohantaviridae*	Ribavirin	[101,102]
	Rift Valley fever virus	*Phenuiviridae/Phlebovirus*	Favipiravir	[103]
	Lymphocytic choriomeningitis virus	*Arenaviridae/Mammarenavirus*	5-Fluorouracil	[74,104]
	Ebola virus	*Filoviridae/Ebolavirus*	Favipiravir	[105]
	Marburg virus	*Filoviridae/Marburgvirus*	Favipiravir	[105]

## 5. Mutagenic Effects of Ribavirin

Ribavirin [1-(β-d-ribofuranosyl)-1,2,4-triazole-3-carboxamide] (Figure 2) exhibits broad-spectrum antiviral activity against DNA- and RNA-based viruses. Despite being widely used in clinics for almost five decades, its efficacy has only been established for chronic hepatitis C virus infection, chronic hepatitis E virus infection in transplant recipients, and respiratory syncytial virus infection in infants and immunocompromised elderly patients. In addition, it is used to treat infections causing hemorrhagic fevers (e.g., Lassa fever, Crimean–Congo hemorrhagic fever, and Hantavirus infection), although it has very poor activity against filoviruses, such as Ebola or Marburg viruses [106,107]. 

The mechanism of action of ribavirin has remained largely elusive, probably due to the multiple targets of the drug [108,109]. These mechanisms include host-targeted effects, such as the inhibition of inosine monophosphate dehydrogenase (IMPDH) by ribavirin 5′-monophosphate (RMP), host immune response modulation (including the regulation of interferon-stimulated gene expression), and inhibition of translation initiation, through ribavirin binding to the translation initiation factor 4E (eIF4E) or enzymes responsible for RNA cap synthesis [110]. Interestingly, the lack of viral RNA capping triggers the antiviral host immune response by the recognition of a foreign viral RNA. IMPDH plays a key role in guanine nucleotide biosynthesis by catalyzing the conversion of inosine 5′-monophosphate into xanthine 5′-monophosphate, an intermediate in the de novo synthesis of guanosine. IMPDH regulates intracellular GTP pools necessary for RNA synthesis, and this could explain the activity of ribavirin against both DNA and RNA viruses [111]. However, nucleotide pool alterations due to IMPDH inhibition have a relatively small effect on the increased mutation frequencies observed during ribavirin treatments, as demonstrated for foot-and-mouth disease virus infections in cell cultures [75].

Viral replication is also a target of ribavirin. Thus, viral RNA-dependent RNA polymerases can be inhibited directly by ribavirin 5′-triphosphate (RTP) or can be incorporated (as RMP) into the viral genome, leading to viral mutagenesis [108]. Ribavirin does not have modifications in its sugar moiety and it is not clear how RMP incorporation inhibits RdRp. Ribavirin decreases viral RNA synthesis in infected cells, while in vitro studies have also demonstrated that the drug inhibits the polymerases of influenza A virus, hepatitis C virus, and vesicular stomatitis virus [112]. Studies carried out with poliovirus RdRp showed that RMP incorporation occurs opposite both template cytidine and uridine template residues [69,113]. Virological studies with poliovirus showed that ribavirin-mutagenized genomes had a 600-fold increase in G→A and C→U transition mutations [69]. C→U mutations would be a consequence of the incorporation of RTP as a GTP analog during negative-strand RNA synthesis. The effects of ribavirin as a lethal mutagen have been extensively studied in hepatitis C virus [74,75,76,77,78], but have also been demonstrated for other RNA viruses (Table 2).

**Figure 3:**
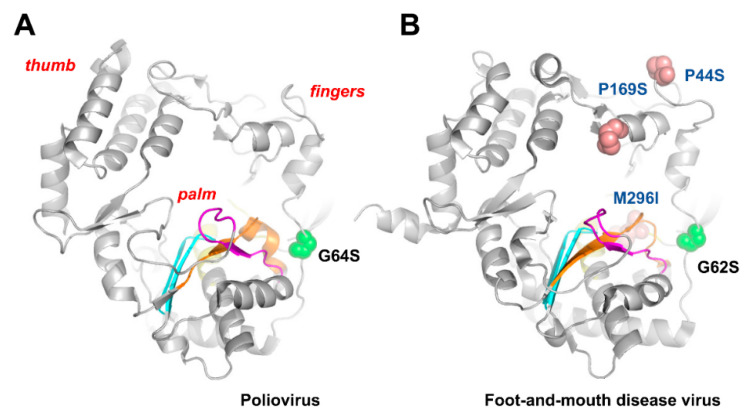
Poliovirus (**A**) and foot-and-mouth disease virus (**B**) RdRp structures showing the location of motifs A–D and residues relevant for ribavirin binding and resistance. Locations of motifs A, B, C, and D are shown in orange, yellow, blue, and magenta, respectively [34,121]. Spheres are used to represent the location of relevant amino acid substitutions. Crystal structures were taken from PDB files 2ILY (poliovirus RdRp) and 1U09 (foot-and-mouth disease virus RdRp).

The early studies with poliovirus suggested that the virus could develop resistance to ribavirin. However, the analysis of the polymerase-coding sequence of virus grown in the presence of 0.8 mM ribavirin showed that conserved motifs C–E remained unchanged, while some variability was observed at motifs A and B [114]. The passage of poliovirus in cell cultures in the presence of ribavirin led to the selection of G64S. This mutation is located at the fingers subdomain of the viral RdRp (Figure 3A). Ribavirin-resistant poliovirus displays increased fidelity of RNA synthesis in the absence of ribavirin, as well as increased survival in the presence of ribavirin and in the presence of 5-azacytidine [115].

Unexpectedly, the equivalent substitution in the RNA polymerase of foot-and-mouth disease virus (i.e., G62S) was never selected when the virus was passaged in the presence of ribavirin and was not detected as a minority variant in the mutant spectra of the virus that replicated in the absence or presence of ribavirin or other mutagenic agents [75,77]. Instead, P44S, P169S, and M296I in the RdRp of foot-and-mouth disease virus (serotype C) were shown to confer different levels of ribavirin resistance [77,78,116] (Figure 3B), while selection studies with the drug using hepatitis C virus replicons selected for P415Y in the thumb subdomain of the polymerase [117]. However, this substitution did not lead to treatment failure in infected patients treated with interferon and ribavirin, or to ribavirin resistance in cell culture assays [118,119]. More recently, Mejer et al. [120] showed, in a hepatitis C virus genotype 3a cell-culture-adapted strain, that certain combinations of mutations selected in patients treated with ribavirin (e.g., D148N/I363V, A150V/I363V, and T227S/S183P) conferred resistance, possibly by increasing the overall fidelity of the viral polymerase as a putative mechanism for ribavirin resistance. Although the structural basis of ribavirin resistance is still uncertain, many of the involved mutations are located at the periphery of the nucleotide entry site in the predicted polymerase structure.

Apart from the structural constraints and mechanisms affecting ribavirin incorporation and nucleotide selectivity by the viral RdRp, it should be noted that viral fitness might be an important factor contributing to the viral response to lethal mutagenesis. Cell culture studies with hepatitis C virus showed that high-fitness viral quasispecies showed resistance to ribavirin and favipiravir without modifying the mutation-type bias typical of those mutagens, probably by limiting the expansion of their mutational spectra [122]. Although ribavirin has been extensively used for various decades, its interaction with the host cell machinery results in poor selectivity and toxicity, yielding undesirable side effects, including severe anemia.

## 6. Favipiravir as a Lethal Mutagenesis Agent

Favipiravir (6-fluoro-3-oxo-3,4-dihydropyrazine-2-carboxamide, T-705) (Figure 2) is an antiviral drug used in Japan to treat influenza. Favipiravir is a prodrug whose active form (favipiravir-ribofuranosyl-5′-triphosphate) mimics both guanosine and adenosine as substrates of viral RdRPs. It has a broad-spectrum antiviral effect, and, apart from influenza virus, favipiravir is also able to inhibit the replication of flavi-, alpha-, filo-, bunya-, arena-, noro-, and other RNA viruses, including neglected and (re)emerging viruses for which no antiviral therapy is currently available [123,124].

Enzyme kinetic analysis with influenza A virus RdRp demonstrated that favipiravir-ribofuranosyl-5′-triphosphate inhibited the incorporation of ATP and GTP in a competitive manner [125,126]. In addition, the incorporation of favipiravir into the nascent RNA strand as a purine nucleotide analog inhibited its further extension [125]. Cell culture studies with influenza A H1N1 viruses showed that favipiravir treatment produced an increased number of G→A and C→T mutations, suggesting that the favipiravir-ribofuranosyl-5′-triphosphate base pairs with either cytosine or uracil [127]. The increased mutation frequency is also dose-dependent, demonstrating that favipiravir is also a lethal mutagen. Therefore, the combination of ambiguous base-pairing with low discrimination of favipiravir-ribofuranosyl-5′-triphosphate is a key factor contributing to the mutagenic effect of favipiravir. 

Evidence of the mutagenic effects of favipiravir in infected animals was reported after studying norovirus infections in a mouse model [80]. Viral RNA isolated from treated animals showed reduced infectivity, while a five- to six-fold increase in mutation frequency was obtained after the sequence analysis of individual molecular clones isolated from populations subjected to four passages in the presence of a drug concentration of 200 μM. Interestingly, these increases were higher than those obtained with ribavirin in parallel experiments (estimated at around three-fold). Favipiravir treatments led to a slight increase in the A→G and U→C transition rates in these assays. The study by Arias et al. [80] constitutes a proof of concept for lethal mutagenesis in vivo, supporting antiviral therapies based on mutagenic compounds at the clinical level. Table 2 shows several examples of viruses where favipiravir has been successfully used to extinguish the virus through lethal mutagenesis, with a concomitant excess of G→A and C→U transitions in the mutant spectrum of preextinction virus populations [91,92,95,103], although these preferences were not always predominant, as reported for favipiravir extinction experiments with foot-and-mouth disease virus [79] and West Nile virus [84].

The heterotrimeric influenza virus polymerase, containing the PA, PB1, and PB2 proteins, catalyzes viral RNA replication and transcription in the nucleus of infected cells. Studies with H1N1, H3N2, and H7N9 strains of influenza A virus showed that K229R in the catalytic PB1 subunit confers favipiravir resistance while impairing viral replication [128]. K229R reduced the mutagenic effect of favipiravir at a cost to growth, but this effect could be alleviated by P653L in the PA subunit. The combination of both mutations led to a virus that was 30-fold less susceptible to favipiravir relative to the wild-type virus and not impaired in replication kinetics [128]. Although the clinical relevance of these mutations is unclear, studies with ferrets have shown the transmissibility of the favipiravir-resistant strains in vivo [129]. 

Favipiravir resistance has also been mapped in the RNA polymerases of chikungunya virus (i.e., K291R) [130] and enterovirus 71 (i.e., S121N) [131], although the antiviral effects were assessed in replication assays and the mutation frequencies were not determined. Favipiravir has been approved for COVID-19 treatment in Russia, but there is no evidence of resistance so far. Based on the structural information of SARS-CoV-2 RdRp bound to favipiravir-ribofuranosyl-5′-triphosphate [132] and the variability observed in circulating viruses, a number of residues have been predicted as potentially involved in favipiravir resistance [133]. However, the phenotypic effects on resistance caused by the proposed substitutions are still being investigated. 

One of the major limitations to the use of favipiravir is its relatively low bioavailability, resulting in relatively low plasma concentrations of the drug. This is apparently due to its short half-life caused by rapid renal elimination. Strategies to increase its potency are needed, particularly considering its strong antiviral effect against different viruses and in different animal models (most notably, mice, Guinea pigs, and non-human primates) (reviewed in [112]). Despite these limitations, Clinicaltrials.gov (accessed on 10 April 2022) currently includes more than one hundred clinical trials testing the efficacy of favipiravir alone or in combination with other drugs (https://clinicaltrials.gov/, accessed on 10 April 2022). Most of these trials (>85%) evaluate the efficacy of favipiravir as a drug against SARS-CoV-2, although there are trials where the compound has been tested against influenza, Ebola, or Lassa virus infections. 

## 7. Molnupiravir as a Broad-Spectrum Antiviral Drug Effective against SARS-CoV-2

Molnupiravir (EIDD-2801, MK-4482) is the isopropylester prodrug of the nucleoside derivative β-d-*N*^4^-hydroxycytidine (NHC, EIDD-1931) (Figure 2). The triphosphorylated form of β-d-*N*^4^-hydroxycytidine is a substrate of RdRps and interferes with viral replication. Molnupiravir is a broad-spectrum antiviral compound that inhibits multiple viruses in cell culture. Examples of molnupiravir-susceptible viruses are Chikungunya virus, Venezuela equine encephalitis virus, respiratory syncytial virus, hepatitis C virus, norovirus, influenza A and B viruses, Ebola virus, and human coronaviruses [94,96,99]. Currently in phase 2/3 clinical trials, molnupiravir has been approved in Britain for the clinical treatment of SARS-CoV-2 infections. In cell culture assays, SARS-CoV-2 (and the related Middle East respiratory syndrome (MERS) coronavirus) were found to be effectively inhibited by submicromolar concentrations of molnupiravir [96,134]. Evidence of its mutagenic effect was also noted in experiments with SARS-CoV-2 when authors found that β-d-N^4^-hydroxycytidine increased the proportion of G→A while inducing C→U transitions after a single round of infection [96], as well as in MERS coronavirus-infected mice treated with β-d-N^4^-hydroxycytidine [135] and influenza virus in animal models [99]. 

**Figure 4:**
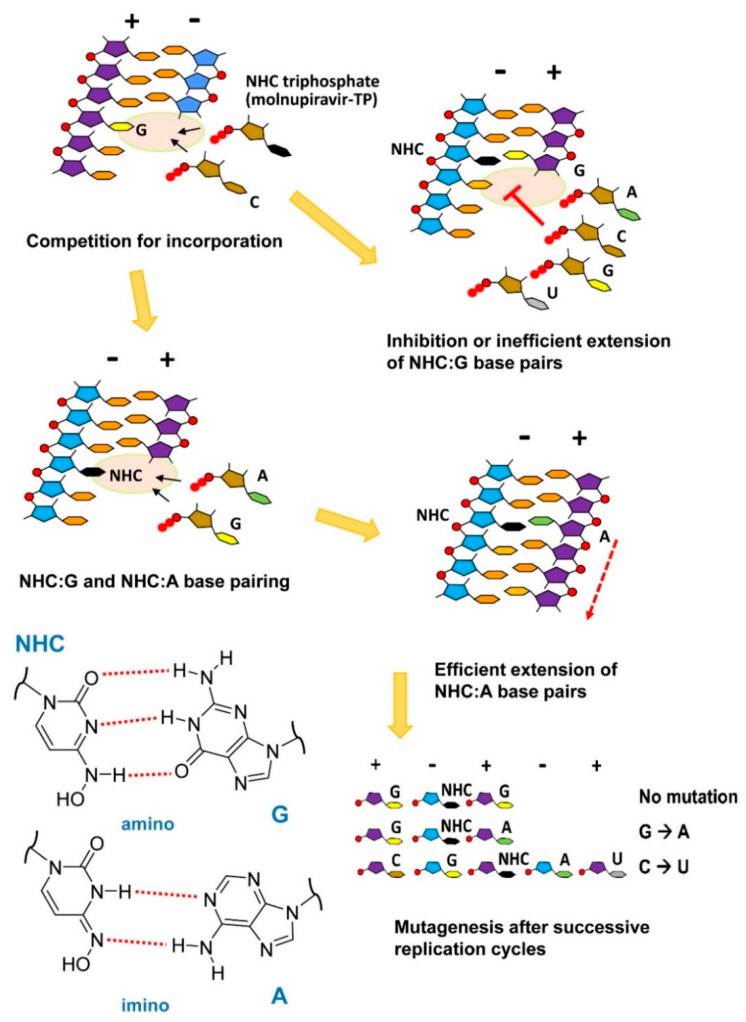
Proposed model for the antiviral mechanism of molnupiravir based on β-d-N^4^-hydroxycytidine (NHC)-induced mutagenesis and the inhibition of RNA synthesis. Tautomeric forms and the base pairing of NHC are shown in the lower-left panel. Illustration based on the experimental data reported by Gordon et al. [136] and Kabinger et al. [137], and adapted from ref. [146].

Two papers published in 2021 described the biochemical and structural bases of how molnupiravir impairs the fidelity of SARS-CoV-2 replication and provokes error catastrophe [136,137]. Both labs found that β-d-N^4^-hydroxycytidine triphosphate competes effectively with cytidine triphosphate (CTP) for incorporation into the nascent RNA. Molnupiravir is then effectively used as a template in the next round of viral RNA synthesis. β-d-N^4^-hydroxycytidine (NHC) forms base pairs with A and G due to the tautomerization of the cytosine analog [138]. The formation of NHC:G mispairs could lead to RNA synthesis inhibition [136], but NHC:A base-pairing induces mutagenesis leading to increased G→A and C→U transition frequencies (Figure 4), in agreement with results obtained in vitro and in vivo [96,135]. 

High-resolution cryo-EM structures showed that SARS-CoV-2 RdRp can accommodate NHC:G and NHC:A mispairs without introducing major distortions in the enzyme’s active site or the nucleic acid scaffold [137]. Although the prevalence of G→A and C→U transition mutations can be explained by the incorporation of β-d-N^4^-hydroxycytidine in the template strand [136], the low prevalence of A→G transitions found by Sheahan et al. [135] could be explained if we assume that β-d-N^4^-hydroxycytidine triphosphate also competes weakly with UTP during RNA polymerization [137]. 

The biochemical experiments described above were carried out with the RdRp holoenzyme, without the intervention of the exonuclease protein (nsp14) found in the SARS-CoV-2 replicase-transcriptase complex. However, previous studies with the related complexes of murine hepatitis virus and MERS coronaviruses revealed that β-d-N^4^-hydroxycytidine is a potent inhibitor of its exonuclease activity [96].

The efficacy of molnupiravir against SARS-CoV-2 has been observed in animal models and humanized mice [135,139], while the administration of β-d-N^4^-hydroxycytidine to infected ferrets prevented SARS-CoV-2 transmission in untreated and uninfected animals [140]. In October 2021, Merck Sharp & Dohme reported results from the interim analysis of a phase 3 clinical trial showing that molnupiravir reduced the risk of admission to hospital or death by approximately 50% in non-hospitalized adults with mild to moderate COVID-19 and at risk of poor outcomes, although the efficacy was reduced to roughly 30% when the study was completed [141]. The interim analysis results led to the approval by the British regulatory agency of Lagevrio (molnupiravir) in November 2021. In December 2021, the U.S. Food and Drug Administration (FDA) also granted an emergency use authorization to molnupiravir for use in certain populations where other treatments are not feasible. Further evidence of molnupiravir’s efficacy has been obtained in a phase 2a clinical trial, where individuals receiving an 800 mg dose of the drug reduced the viral RNA more rapidly than those receiving a placebo while eliminating the infectious virus in nasopharyngeal swabs [142]. In addition, molnupiravir retains efficacy in cell culture and animal models against the original SARS-CoV-2 strains, as well as the later emerging variants of concern alpha (B.1.1.7), beta (B.1.351), gamma (P.1), delta (B.1.617.2), and omicron (B1.1.529) [143,144,145]. An added benefit of molnupiravir is its high genetic barrier to resistance [96,99].

A synergistic effect has been reported for molnupiravir and favipiravir in the hamster model [147]. The synergy was achieved at doses where either drug had demonstrated antiviral activity. However, so far, favipiravir has shown antiviral activity in the hamster model, but not in larger animal models or in humans, due to its rapid turnover, and better ways of administration are required to improve the pharmacokinetics of the drug. Interestingly, in a recent study, Schultz et al. [148] demonstrated a strong synergistic effect of pyrimidine biosynthesis inhibitors (most notably DHODH inhibitors, such as brequinar or BAY-2402234) with molnupiravir in inhibiting SARS-CoV-2 infection in vitro and in vivo against emerging strains of SARS-CoV-2. These findings argue in favor of the clinical testing of molnupiravir in combination with different drugs as part of current efforts aimed to fight the current pandemic. 

Despite evidence showing the efficacy of molnupiravir, there are still concerns about its use. Getting molnupiravir to patients in a timely way is likely to be problematic, and its mutagenic potential is a matter of concern. β-d-*N*^4^-hydroxycytidine can be metabolized by the host cell to the 2′-deoxyribonucleoside form by the ribonucleoside reductase and then incorporated into the host cell’s DNA. The mutagenic effect of β-d-*N*^4^-hydroxycytidine has been shown in animal cell cultures [68]. However, these findings are difficult to confirm in animal models. 

Candidate drugs are subjected to genotoxicity assays with somatic cells to determine the possibility of mutations or chromosomal abnormalities that could pose a risk to human health. Pig-a, Big Blue^®^ (*cII* locus), or Muta^TM^Mouse *LacZ* are examples of well-established genotoxicity assays. The reported genotoxicity profile for molnupiravir was negative with the Big Blue^®^ (*cII* locus) assay and inconclusive with Pig-a [149]. The Pig-a mutation assay is a flow cytometry-based assay developed for cells of peripheral blood (e.g., red blood and white blood cells) that harbor inactivating mutations in the phosphatidyl inositolglycan class A gene, linked to chromosome X [150]. Furthermore, negative in vivo animal mutagenicity cannot completely exclude a genetic risk to patients in clinical trials. Therefore, the human genetic risk of treatment needs to be carefully evaluated for molnupiravir and other mutagenic nucleosides, since antiviral lethal mutagens have the potential of permanently modifying the genomes of treated patients while causing human teratogenicity or embryotoxicity [151].

## 8. Future Directions and Challenges

The development of lethal mutagens faces several challenges. Most notably, the cell toxicity needs to be very low, particularly in relation to their carcinogenic potential. Similar to the case of other antivirals, drug resistance is a fundamental issue, not only because it could lead to drug inactivation, but also because a mutagen could spur the emergence of novel virus variants with potentially increased pathogenicity and transmissibility. Attempts to design and develop bona fide lethal mutagens (such as KP1212, for example) have not been successful and issues related to their efficacy and selectivity need to be addressed. However, lethal mutagens, such as ribavirin or favipiravir, were approved for clinical use based on their antiviral efficacy before the demonstration of their mutagenic effect. In the case of ribavirin, the drug has been used for decades to treat chronic hepatitis C, although with limited efficacy and adverse side effects. The mutagenic effect of ribavirin has been demonstrated with several viruses, including hepatitis C virus (Table 2). However, ribavirin has multiple targets, including enzymes and processes occurring in the host cell, and its side effects seem to be unrelated to mutagenicity.

Interestingly, favipiravir and molnupiravir act mainly by driving viruses to error catastrophe. Both compounds have broad-spectrum activity and show some resilience to drug resistance. Molnupiravir shows a very high genetic barrier to resistance and the selection of mutants conferring drug resistance has not been successful. However, learning from experience with HIV and other viruses [52,152], combination therapies should be the choice for a most efficient treatment and, in the case of COVID-19, further studies will be necessary to test the efficacy of molnupiravir combined with remdesivir (Veklury) or the protease inhibitor nirmatrelvir (Paxlovid). Future research efforts should also concentrate on finding or designing novel, more specific mutagenic agents acting on viral polymerases, suitable for combination therapies in order to avoid the selection of escape mutants. 

Concerns about the potential genotoxicity of lethal mutagens are justified and have to be carefully addressed. However, in acute infections leading to COVID-19 or flu, the relatively short incubation time limits the extent of the medication and, therefore, the exposure to the mutagenic compounds. Reliable assessment of mutagenicity and carcinogenicity is still complex, since the results obtained in current tests (usually carried out in mice or rats) are difficult to confirm in primate models or humans. Another issue that is not always considered in genotoxicity assays is the impact of the drug exposure time. This could be short in acute viral infections, but long and probably hazardous in chronic infections. Finally, the prescription of mutagenic nucleosides should take into account long-term pharmacovigilance. A registry of patients treated with those drugs would be very helpful for a long-term follow-up of potential adverse effects, including genetic, carcinogenic, teratogenic, and embryotoxic damage.

## Data Availability

Not applicable.
